# Dynamics of EGFR mutations in plasma recapitulates the clinical response to EGFR-TKIs in NSCLC patients

**DOI:** 10.18632/oncotarget.19139

**Published:** 2017-07-10

**Authors:** Liwen Xiong, Shaohua Cui, Jingyan Ding, Yun Sun, Longfu Zhang, Yizhuo Zhao, Aiqin Gu, Tianqing Chu, Huimin Wang, Hua Zhong, Xin Ye, Yi Gu, Xin Zhang, Min Hu, Liyan Jiang

**Affiliations:** ^1^ Department of Pulmonary Medicine, Shanghai Chest Hospital, Shanghai Jiao Tong University, Shanghai, China; ^2^ IMed Asia, AstraZeneca, Shanghai, China; ^3^ Department of Pulmonary Medicine, Zhongshan Hospital, Shanghai Fudan University, Shanghai, China

**Keywords:** epidermal growth factor receptor (EGFR), EGFR tyrosine kinase inhibitors (EGFR-TKIs), circulating tumor DNA (ctDNA), droplet digital PCR (ddPCR), non-small cell lung cancer (NSCLC)

## Abstract

**Objectives:**

Genomic profiling using plasma cell-free DNA (cfDNA) represents a non-invasive alternative to tumor re-biopsy, which is challenging in clinical practice. The feasibility of dynamically monitoring epidermal growth factor receptor (EGFR) mutation status using serial plasma samples from non-small cell lung cancer (NSCLC) patients treated by tyrosine kinase inhibitors (TKIs) and its application in tracking clinical response and detection of resistance were investigated.

**Patients and methods:**

Forty-five NSCLC patients with EGFR mutation-positive pre-TKI plasma and at least two post-TKI plasma collections were recruited to this study. EGFR mutations including L858R, exon 19 deletion (19-del) and T790M were analyzed using droplet digital PCR (ddPCR) in longitudinally collected plasma samples.

**Results:**

We observed a significant reduction in plasma EGFR mutation abundance during the first two-month of TKI treatment. Acquiring of secondary T790M gatekeeper mutation or completed “loss” of EGFR mutations represented two major categories of resistance profiles. Moreover, we demonstrated that levels of plasma EGFR mutations highly correlated with changes of tumor diameter as determined by radiographic imaging, or development of new lesions. In a subset of patients, we further showed that reappearance of EGFR mutations could be detected in plasma up to 5 months ahead of progressive disease (PD), suggesting an early detection of drug resistance.

**Conclusions:**

Our findings suggest that genomic analysis using plasma cfDNA may offer an effective approach to monitor clinical response and emergence of resistance.

## INTRODUCTION

Non-small cell lung cancer (NSCLC) is the leading cause of cancer-related death worldwide [1]. Epidermal growth factor receptor (EGFR) activating mutations such as L858R and exon 19 deletion (19-del) account for 30-60% of NSCLC cases in Asia [2–4]. NSCLC patients with EGFR L858R or 19-del mutations can benefit from treatment of tyrosine kinase inhibitors (TKIs) [5–12]. However, resistance will eventually occur with ∼60% of cases resulting from the acquired secondary EGFR T790M gatekeeper mutation, and the rest from bypass tracks such as ERBB2 and MET amplification, BRAF and PIK3CA mutations, or SCLC transformation [13–17]. To dynamically monitor the emergence of resistance and adjust treatment regimen accordingly, it is important to carry out molecular characterization on serial disease progression specimens. However, tumor re-biopsies are challenging to obtain in clinical practice due to invasiveness of the procedures and potential complications [18]. Moreover, genomic information acquired from a single biopsy only provides a spatially and temporally limited snapshot of a tumor and might fail to reflect its heterogeneity and evolution [19].

Cancerous tissues are able to release DNA into circulation through apoptosis or necrosis, which is called circulating tumor DNA (ctDNA) and comprises a fraction of plasma cell-free DNA (cfDNA). Back in very early days, Leon *et al* [20] already reported high concentrations of cfDNA in the circulation of cancer patients, and Stroun *et al* [21] demonstrated the presence of DNA fragments released from heterogeneous tumor cell clones in the circulation. ctDNA carries tumor-specific genomic alterations including point mutations, copy number fluctuations and structural rearrangements, and can be detected by various technologies either qualitatively or quantitatively. Thus liquid biopsy using cfDNA represents an effective complement to tumor re-biopsies thanks to its non-invasive, easy-to-access features, which allow for longitudinal monitoring of the disease [22, 23].

Previously we have developed droplet digital PCR (ddPCR) assays for highly sensitive and quantitative measurement of EGFR mutations in plasma cfDNA [24, 25]. In this study, we conducted a two-center retrospective study in EGFR mutation-positive Chinese NSCLC patients who were treated with TKIs, and evaluated the correlation of their plasma EGFR mutation profiles with changes of tumor diameter and development of new lesions. Our data suggested that dynamics of plasma EGFR mutations might serve as a surrogate biomarker to monitor clinical response and emergence of resistance.

## RESULTS

### Patients’ clinical characteristics and plasma sample collection

**Figure 1:**
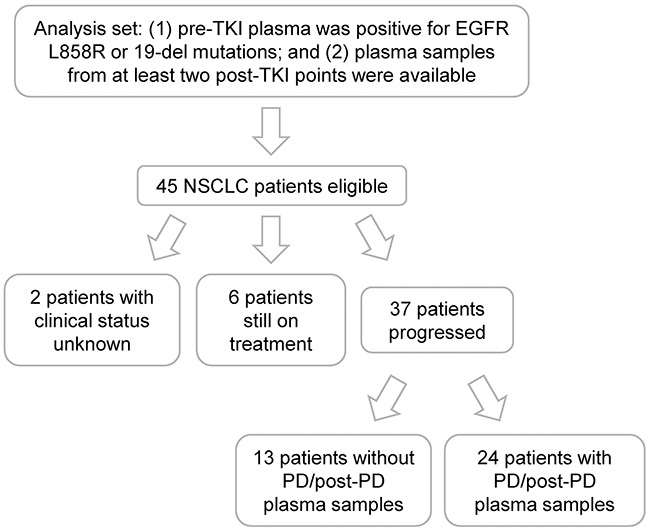
Patient recruitment flow chart

NSCLC patients were included in the analysis set if (1) their pre-TKI plasma was positive for EGFR L858R or 19-del mutations; and (2) they had plasma samples from at least two post-TKI points (Figure 1). In total, 45 patients were eligible and their clinical characteristics were described in Table 1. The median age for this cohort was 58 with the range of 37-77 and over half were female. Adenocarcinoma, stage IV, non-smoker, and first-line gefitinib treatment occupied the majority of the population. Twenty-eight patients had pre-TKI plasma samples with 19-del, 16 with L858R, and one with L858R & 19-del dual mutations. Concordant plasma and tissue EGFR mutation testing results were found in 42 patients (Supplementary Table 1). By the data cut-off date, 6 patients were still on treatment and 37 reached progressive disease (PD) (Figure 1). Serial plasma samples were taken throughout TKI treatment for each patient with most post-TKI samples collected at 1- and 3-month (Supplementary Figure 1). A total of 210 plasma samples were used for analysis of EGFR mutations.

**Table 1:** Clinical characteristics of 45 NSCLC patients and their EGFR mutation type(s) in pre-TKI plasma and tumor specimens

			%
**Age**	**Median (range)**	58 (37-77)	
**Gender**	**Men/women**	19/26	42.2/57.8
**Histology**	**Adenocarcinoma/unknown**	44/1	97.8/2.2
**Stage**	**IIIB/IV/unknown**	6/38/1	13.3/84.4/2.2
**Smoking**	**Yes/No/unknown**	9/35/1	20/77.8/2.2
**EGFR-TKI treatment**			
Gefitinib/Icotinib		30/15	66.7/33.3
1st line/2nd line or after		35/10	77.8/22.2
**Pre-TKI plasma EGFR mutation type(s)**			
19-del		28	62.2
L858R		16	35.6
19-del & L858R		1	2.2
**Pre-TKI tissue EGFR mutation type(s)**			
19-del		27	60.0
L858R		17	37.8
19-del & L858R		1	2.2

### Dynamic monitoring of EGFR mutations in plasma samples from 45 NSCLC patients as a pool

Dynamic detection of EGFR mutations during TKI treatment from baseline to post-PD for the 45 patients was performed (Supplementary Figure 2; Supplementary Table 2). Overall, the positive rate for L858R/19-del decreased along the treatment span. In the meanwhile, the resistance mutation T790M arose gradually with time, and occurred simultaneously with L858R or 19-del in majority of cases. Moreover, T790M could be detected in plasma by ddPCR prior to clinical PD.

### Investigation of resistance profiles using plasma samples

**Figure 2:**
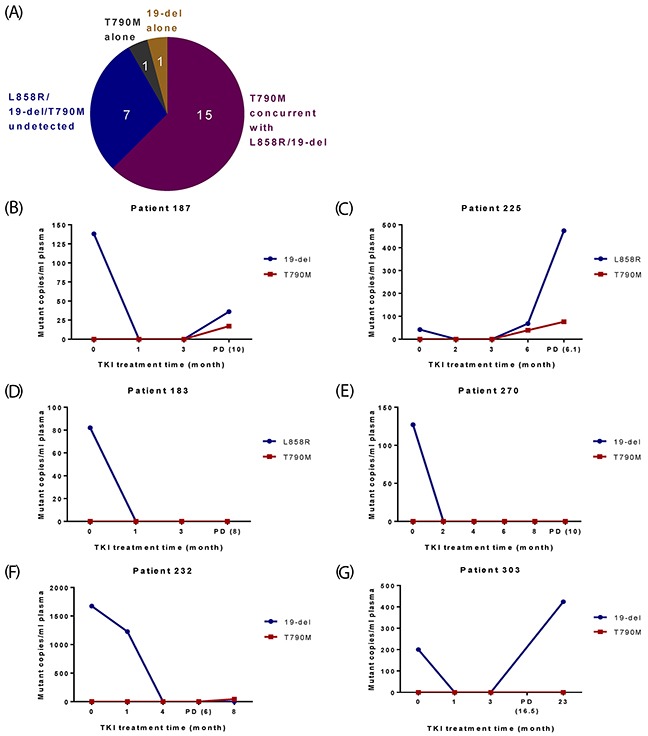
Profiles of EGFR mutations in plasma for 24 NSCLC patients who progressed **(A)** Categories of EGFR mutation status in plasma at/after PD. **(B-G)** Representative results from patients in different categories. X-axis is TKI treatment time and Y-axis is EGFR mutant copies per ml of plasma. **(B-C)** Two patients initially carried 19-del/L8589R and eventually developed T790M concurrently with 19-del/L858R. **(D-E)** Two patients initially carried 19-del/L858R, but “lost” EGFR mutations at PD. **(F)** One patient showed T790M only after PD. **(G)** One patient carried 19-del alone after PD.

Twenty-four patients progressed with their PD/post-PD plasma samples available for EGFR mutation testing (Figure 1). Four categories of resistance profiles were identified - 15 of them developed T790M concurrent with L858R or 19-del; seven completely “lost” EGFR mutations at/after PD; one had T790M alone and one had 19-del alone (Figure 2A-2G; Supplementary Table 3). Additionally, T790M together with L858R or 19-del were detected in three patients who were lack of PD/post-PD plasma samples or did not yet progress (Supplementary Table 3).

### Correlation of plasma EGFR mutation abundance with clinical response and early detection of resistance

A key question to be addressed in this study is whether plasma EGFR mutation abundance could serve as a surrogate biomarker to monitor clinical response and emergence of resistance. Therefore, we collected tumor-imaging data whenever available for all the patients whose plasma samples were EGFR mutation-positive at ≥1 post-TKI points. Sixteen patients met this criteria with evaluable CT scans.

**Figure 3:**
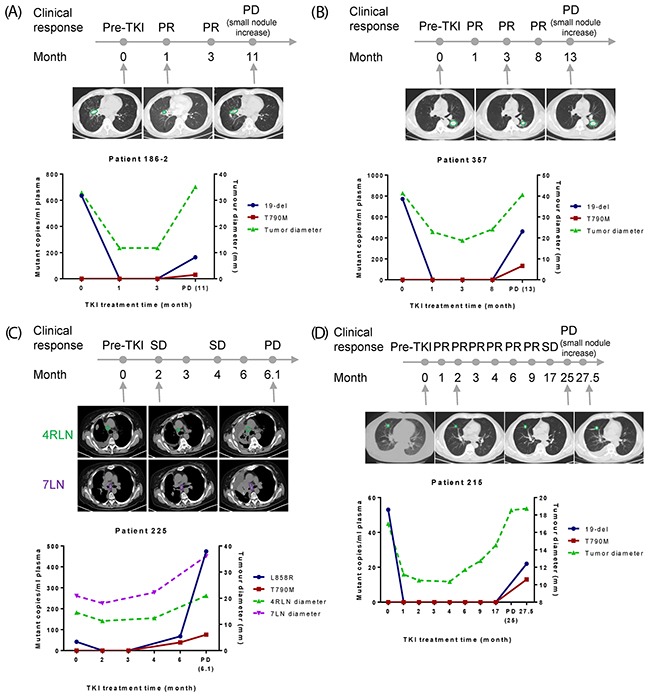
Correlation of EGFR mutation abundance in plasma with clinical imaging data X-axis is TKI treatment time and Y-axis is EGFR mutant copies per ml of plasma (left) or tumor diameter (right). **(A-D)** Results from four patients to show nice correlation between plasma EGFR mutation abundance and tumor/lymph node diameter. CT scans of selected time points were shown and lesions were indicated by green or purple circles.

Patient 186-2 (Figure 3A) initially carried plasma 19-del (635 mutant copies/ml) which dropped to the point where the mutation was undetectable after 1-month of TKI treatment. Until 3-month, this patient showed good response to the therapy. However, after 11 months, the patient developed PD with T790M (30 mutant copies/ml plasma) appeared simultaneously with 19-del (164 mutant copies/ml plasma) in the plasma samples. The changes on tumor diameter showed the same dynamic pattern as the plasma EGFR mutation abundance.

Patient 357 (Figure 3B) initially carried plasma 19-del (772 mutant copies/ml). This patient showed the best response at 3-month and developed PD with the rise of T790M and 19-del (19-del: 462 mutant copies/ml plasma; T790M: 133 mutant copies/ml plasma), which was consistent with chest CT results showing that the size of both primary tumor and small nodules increased.

Patient 225 (Figure 3C) initially was plasma L858R positive (42 mutant copies/ml plasma). This patient responded well to TKI by 3-month. At 6-month, T790M together with L858R emerged, and 3 days later, increased dramatically (L858R: 474 mutant copies/ml plasma; T790M: 76 mutant copies/ml plasma). The chest CT results from lymph nodes also showed clinical PD at this point.

Patient 215 (Figure 3D) initially carried plasma 19-del (53 mutant copies/ml plasma). This patient showed disease control up to 17-month and developed PD at 25-month. Both T790M and 19-del (19-del: 22 mutant copies/ml plasma; T790M: 13 mutant copies/ml plasma) were detected at 27.5 month. The plasma EGFR mutation abundance correlated well with tumor diameter.

The rise of both activating mutations and T790M was detected at PD and onwards for five additional patients (Supplementary Figure 3A-3E), which was consistent with occurrence of new lesions though primary tumor diameter maintained, suggesting that plasma mutation testing could reflect the changes of tumor burden from the whole body.

**Figure 4:**
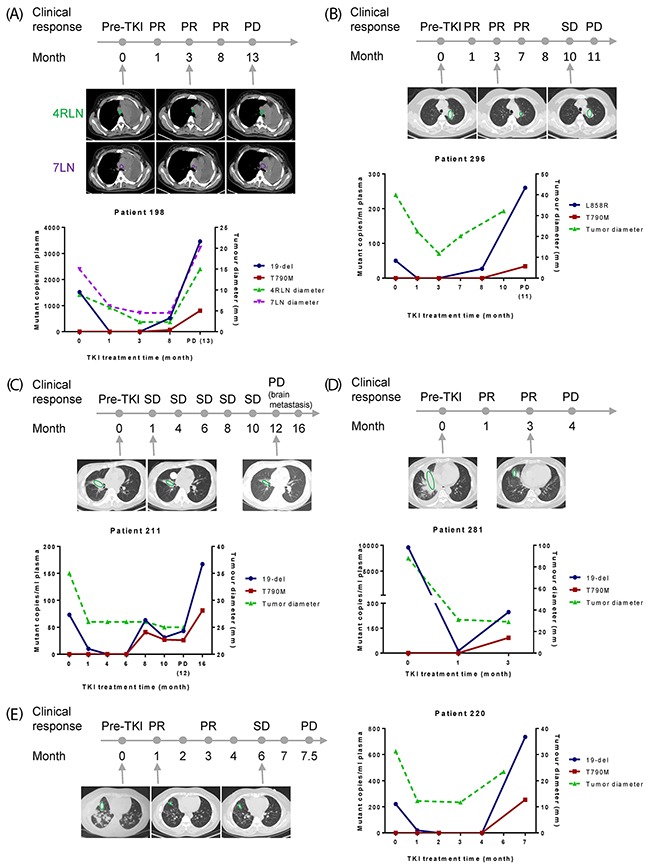
Early detection of resistance by plasma EGFR mutation testing X-axis is TKI treatment time and Y-axis is EGFR mutant copies per ml of plasma (left) or tumor diameter (right). **(A-E)** Results from five patients to show early detection of resistance before clinical PD. For patient 211, PD was defined as development of a new brain metastasis lesion. CT scans of selected time points were shown and lesions were indicated by green or purple circles.

Moreover, not only we observed nice correlation between plasma EGFR mutation abundance and clinical response in the 9 patients mentioned above, but also an early indication of resistance among the other 7 cases (Figure 4A-4E; Supplementary Figure 4A-4B).

Patient 198 (Figure 4A) initially had 19-del (1518 mutant copies/ml plasma) and later showed rise of T790M (67 mutant copies/ml plasma) and 19-del (522 mutant copies/ml plasma) at 8-month, about 5 months before clinical PD. The abundance of both mutations increased along the time span and reached to very high level (T790M: 805 mutant copies/ml plasma; 19-del: 3460 mutant copies/ml plasma) at PD.

Patient 296 (Figure 4B) initially carried plasma L858R (50 mutant copies/ml plasma). This patient responded well to TKI by 3-month. At 8-month, L858R rose again (27 mutant copies/ml plasma), and 3 months later, increased dramatically with T790M at PD (L858R: 260 mutant copies/ml plasma; T790M: 34 mutant copies/ml plasma).

Patients 211 (Figure 4C) showed stable disease up to 10-month of TKI treatment, and 19-del and T790M were detected in plasma from the 8-month of TKI treatment, around 4 months ahead of clinical PD with development of brain metastasis.

Patient 281 (Figure 4D) showed rise of T790M mutation (92 mutant copies/ml plasma) at 3-month post-TKI. One month later, chest CT showed that this patient progressed.

Patient 220 (Figure 4E) showed emergence of T790M (254 mutant copies/ml plasma) concurrent with 19-del two weeks before switching to another therapeutic regimen.

Patients 263 (Supplementary Figure 4A) showed stable disease up to 8-month of TKI treatment. However, the emergence of L858R and T790M mutations were detected in plasma at 7-month post-TKI, around 4 months before clinical PD.

For patient 477 (Supplementary Figure 4B), 19-del and T790M were detected at 10-month post-TKI with the tumor diameter rebounded close to that of pre-TKI point, though PD was not reached yet.

Taken together, our results suggest that plasma ctDNA testing may serve as a surrogate biomarker to monitor clinical response and emergence of resistance.

## DISCUSSION

Dynamic monitoring of the clinical response and detection of drug resistance are critical for patient management and determining the optimal therapeutic regimen. Liquid biopsy has emerged as a powerful tool to reflect changes of the genomic landscape of tumors during the treatment course due to its non-invasive, easy-to-access features [26, 27]. With the advancement of technologies such as amplification refractory mutation system (ARMS), digital PCR and next-generation sequencing (NGS), the clinical values of plasma ctDNA have been more and more manifest to overcome the limitations of tissue testing including tumor heterogeneity and challenges to obtain re-biopsies [28, 29].

Several studies have demonstrated the feasibility of monitoring EGFR mutations in plasma to evaluate the treatment response to TKIs and predict clinical outcome of NSCLC patients [24, 30–32]. Here, we used highly sensitive ddPCR assays to quantify EGFR mutation abundance in longitudinal plasma samples from baseline to PD. Previously, by comparing plasma samples with paired tumor tissues from 86 TKI-naïve NSCLC patients for L858R/19-del mutations and 25 relapsed patients for T790M, we have demonstrated the robustness of our ddPCR assays [24, 25]. The ctDNA testing sensitivity and specificity reached 80.00% and 95.77% for L858R, 81.82% and 98.44% for 19-del, and 81.25% and 100.00% for T790M, respectively. In the current study, our ddPCR assays also achieved concordance of 93.3% (42/45) between tissue and plasma samples.

In order to track EGFR mutation status, we only enrolled NSCLC patients whose pre-TKI plasma samples were either L858R or 19-del positive into our analysis set. Significant reduction in plasma EGFR mutation abundance during the first two-month of TKI treatment was observed, while resistance mutation T790M accompanied by original activating mutations gradually increased with treatment time and peaked at clinical PD and onwards. The simultaneous elevation of T790M and L858R/19-del implicated that the mutations were very likely co-existing in the same tumor cell clones, although mutation testing on the single cell will be required to fully confirm this hypothesis. These observations suggested that dynamic monitoring of plasma EGFR mutation could predict disease progression during the treatment course, especially for T790M-mediated resistance.

The development of acquired resistance to anti-cancer therapy derives from tumor heterogeneity, clonal evolution and selection pressure [33]. The secondary T790M mutation is the major resistance mechanism to EGFR-TKIs in NSCLC patients, accounting for ∼60% of cases [13, 15, 16]. In our study, we identified two major resistance profiles from the 24 progressed patients whose PD/post-PD plasma samples were available. Fifteen patients had T790M mutation concurrent with activating mutations, consistent with previous reports of T790M mutation rate profiled from tumor re-biopsies of TKI-relapsed NSCLC patients [34–36]. The other 7 patients had neither T790M nor activating mutations detectable at/after PD. This may result from underlying tumor heterogeneity and outgrowth of EGFR wildtype subclones, although we cannot exclude the possibility that other EGFR mutation types contributed to the resistance, which was beyond the detection scope of our ddPCR assays. Similar findings have been described for the 3^rd^ generation EGFR-TKIs, where subset of patients showed T790M “loss” after relapse [37, 38].

To explore whether ctDNA could serve as a surrogate biomarker to monitor clinical response and emergence of resistance, we investigated the correlation between plasma EGFR mutation abundance and tumor diameter as measured by CT scans. Although with relatively small sample size, we consistently observed that levels of plasma EGFR mutations were highly correlated with changes of tumor diameter or development of new lesions. Moreover, it is worthwhile to note that in quite a few patients, T790M mutation could arise in plasma up to 5 months ahead of clinical PD, consistent with previous reports [24, 31, 39]. The accuracy of early detection of resistance can be impacted by several factors, including degree of ctDNA release, assay sensitivity, assay coverage of resistance mechanisms, and plasma collection frequency. In this study, we focused on T790M-mediated resistance, and utilized highly sensitive ddPCR assays and serial plasma samples to address this clinical question. Our results together with others have shed the light on the clinical values of liquid biopsy in allowing early detection and intervention of drug resistance. With the advancement of 3^rd^ generation TKIs, it will be of great interest to evaluate in clinical setting whether early intervention of T790M-mediated resistance might offer patients better outcome. Currently a study called “APPLE trial” (ClinicalTrials.gov identifier: NCT02856893) has been designed to evaluate the best sequential treatment strategy with gefitinib and osimertinib in advanced EGFR mutation-positive NSCLC patients, and to understand the value of plasma ctDNA T790M test as a predictive marker for making treatment decisions [40].

There was a trend that progression-free survival (PFS) in patients who had undetectable plasma EGFR mutations at one-month TKI treatment was longer than those with detectable EGFR mutations (median PFS: 11 months, n=29 *vs*. 6 months, n=5) (Supplementary Table 4). Although it did not reach statistical significance due to small sample size, this is consistent with the findings from FASTACT-2 study [41] and suggests that ctDNA could serve as a predictive biomarker for clinical outcome. Previously, EGFR mutation abundance in tumor tissues has been correlated with benefit from TKI treatment for advanced NSCLC [42]. Median PFS in patients with high abundance of EGFR mutations was significantly longer than those with low abundance, and median PFS of patients with low abundance of EGFR mutations was significantly longer than that of those with mutation-negative tumors. However, we did not observe any correlation between baseline plasma EGFR mutation abundance and clinical outcome in this cohort. One of the reasons could be due to heterogeneity in the degree of ctDNA release from tumors across different patients, and thus mutation abundance in plasma may not proportionally reflect that in tumor tissues.

One limitation of ddPCR technology is that it can only detect known mutations. Our ddPCR assays were designed specifically for EGFR L858R, 19-del and T790M mutations, which restricted their usages on other types of genetic alterations. The advancement of NGS technology will further expand the applications of ctDNA mutation testing.

In conclusion, our study demonstrated that plasma EGFR mutation detection by ddPCR is feasible and effective in monitoring treatment response and resistance to EGFR-TKIs in NSCLC patients under certain conditions. The high sensitivity, simple experimental procedure, and fast turnaround time of ddPCR make it a robust tool in clinical practice. Dynamic monitoring of genetic landscapes of tumor evolution in a non-invasive approach has important clinical values in reflecting the clinical response, predicting survival outcome, understanding resistance mechanisms, and guiding precision medicine.

## MATERIALS AND METHODS

### Patients and samples

Patients with advanced NSCLC who received TKI treatment between Apr 2014 and Feb 2017 at Shanghai Chest Hospital, Shanghai Jiao Tong University and Shanghai Zhongshan Hospital were enrolled consecutively. The study was approved by the hospital research ethics committee and all patients had signed the informed consent form. Serial plasma samples were collected at time points of pre-TKI until PD/post-PD for each patient. Patients were included in the analysis set only if their pre-TKI plasma EGFR mutation status was positive, and they had plasma samples of at least two post-TKI points. EGFR mutation status of their primary tumors was determined by ARMS assay at the hospitals. Tumor longest diameter from radiographic examination was measured at each time point by physicians independently, and clinical PD was defined as 20% increase of tumor diameter comparing with baseline and/or appearance of new lesions.

### Plasma isolation and extraction of cell-free DNA (cfDNA)

Patient blood samples were collected in EDTA tubes and centrifuged at 2500x g for 10min. The supernatants were isolated and stored at −80°C until use. cfDNA was extracted from 4ml plasma samples using QIAamp Circulating Nucleic Acid Kit (Catalog No. 55114, Qiagen), according to the manufacturer's instructions. The extracted cfDNA was eluted in 110μL of elution buffer.

### EGFR mutation detection by ddPCR

Development and validation of ddPCR assays for EGFR L858R, 19-del and T790M have been described previously [24, 25]. ddPCR data was analyzed using QuantaSoft 1.7.4 (Bio-Rad). Samples were considered positive if there were ≥2 mutant droplets in the positive area of FAM signal. The experimental procedures of ddPCR assays and the quantification of EGFR mutation abundance (mutant DNA copies per ml of plasma) were described in the supplementary information. The data was plotted by GraphPad Prism 6.

## SUPPLEMENTARY MATERIALS FIGURES AND TABLES


