# Supplementary material for: Dynamics of EGFR mutations in plasma recapitulates the clinical response to EGFR-TKIs in NSCLC patients
Source: Oncotarget. 2017 Jul 10;8(38):63846–56. doi: 10.18632/oncotarget.19139 (PMC5609966; doi:10.18632/oncotarget.19139)
Supplement: Supplementary file 1 [file oncotarget-08-63846-s001.pdf]

Dynamics of EGFR mutations in plasma recapitulates the clinical response to EGFR-TKIs in NSCLC patients

SUPPLEMENTARY MATERIALS

Experimental procedures of ddPCR assays

For L858R and T790M ddPCR assays, the final 20µL of TaqMan PCR reaction mixture contained 1× ddPCR supermix (Catalog No. 1863010, Bio-Rad Laboratories), 900nM of each primer, 450nM of each probe, and up to 50ng DNA templates. Each assembled ddPCR reaction mixture was transferred for automated droplet generation (Bio-Rad) followed by PCR reaction. Thermal cycling conditions were as follows: 10 min incubation at 95°C followed by 45 cycles of 95°C for 15 sec, 60°C for 1 min, and then 4°C hold. For 19-del ddPCR assay, the final 20µL of TaqMan PCR reaction mixture contained 1× ddPCR supermix (Catalog No. 1863010, Bio-Rad Laboratories), E19-del and E2 primers and probe (900nM of each primer, 450nM of each probe), 900nM E19-del PNA and up to 50ng DNA templates. Thermal cycling conditions were as follows: 10 min incubation at 95°C followed by 45 cycles of 95°C for 15 sec, 70°C for 15 sec, 60°C for 1 min, and then 4°C hold. Droplet fluorescence was collected in QX100 droplet reader (Bio-Rad). Human reference genomic DNA (Catalog No. G1471, Promega) and NTC (nuclease-free water) were routinely included as negative controls. DNA from NCI-H1975 cells (harboring L858R and T790M mutations) and NCI-H1650 cells (harboring 19-del mutation), and different cell line DNA dilution with human reference genomic DNA (1:1000 and 1:2500 mutant allele to wild type allele) were routinely included as positive controls. Both controls were used to determine the cut-off for allele calling.

Quantification of EGFR mutant ctDNA fraction for plasma cfDNA

7.3µL from each plasma cfDNA elute was added into above reaction mixture. The number of positive droplets and sample input follow the Poisson distribution. Plasma sample EGFR mutant/wild type DNA input per reaction (*I*, copies per reaction) was calculated with the equation:

I (copies/reaction) = (-LN(1-p)/V)*1000*20.

p: fraction of positive droplets; V: volume of each droplet (0.91nl).

For 19-del assay, *I* (total DNA copies) equals to the copies of EGFR E2 DNA template (VIC signal). For L858R and T790M assays, *I* (total DNA copies) equals to the copies of EGFR mutant and wild type DNA templates (FAM and VIC signal).

Total DNA copies and EGFR mutant DNA copies/ml plasma were calculated with the equations:

Total DNA copies/ml plasma = *I* (total DNA copies)/7.3*volume of cfDNA eluted/volume of plasma;

EGFR mutant DNA copies/ml plasma = *I* (EGFR mutant copies)/7.3*volume of cfDNA eluted/volume of plasma.

The fraction of EGFR L858R or T790M mutant (F1) was calculated as below:

$$F1 = \frac{I\ (\mathrm{FAM})}{I\ (\mathrm{FAM}) + I\ (\mathrm{VIC})}$$

The fraction of EGFR 19-del mutant (F2) was calculated as below:

$$\mathrm{F2} = \frac{I\ (\mathrm{FAM})}{I\ (\mathrm{VIC})}$$

SUPPLEMENTARY FIGURES AND TABLES

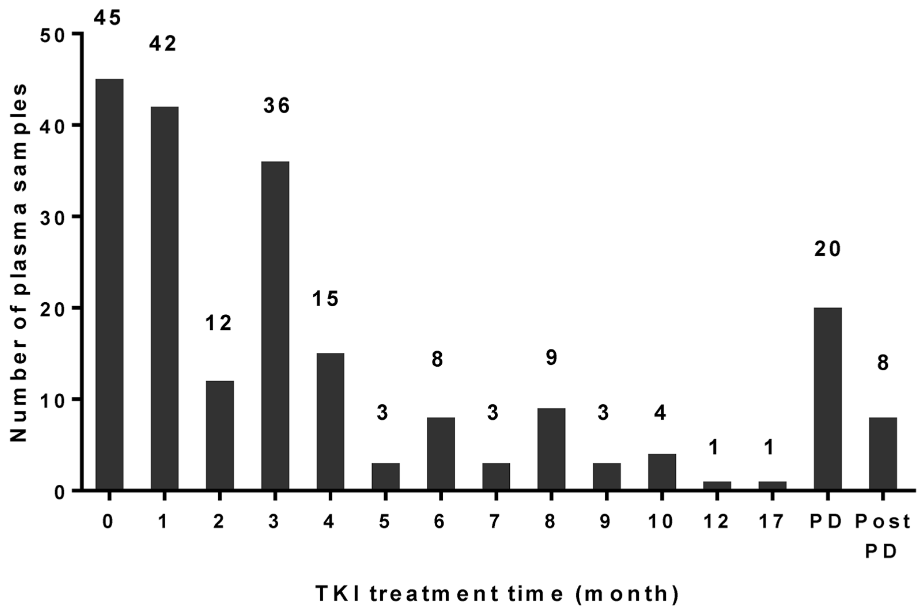

Supplementary Figure 1: Distribution of patient plasma samples collected at pre-TKI, on treatment (1- to 17-month), PD and post-PD time points.

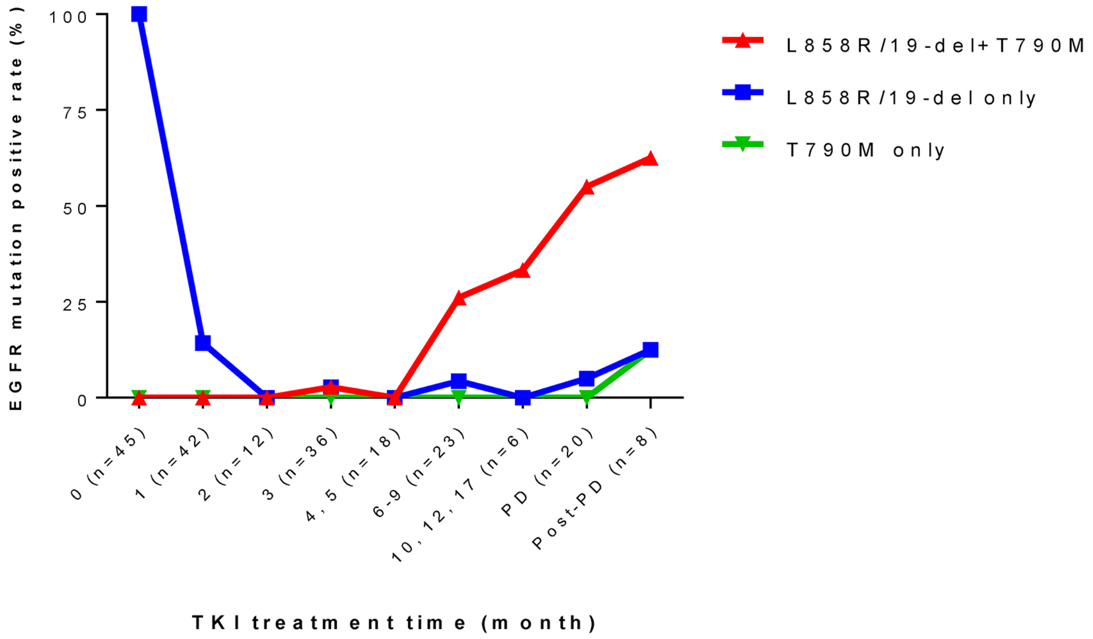

Supplementary Figure 2: Dynamic detection of EGFR mutations in plasma by ddPCR from 45 NSCLC patients as a pool. X-axis is TKI treatment time and Y-axis is the percentage of plasma samples positive for EGFR mutations. “n” in the x-axis is the sample size for each time point.

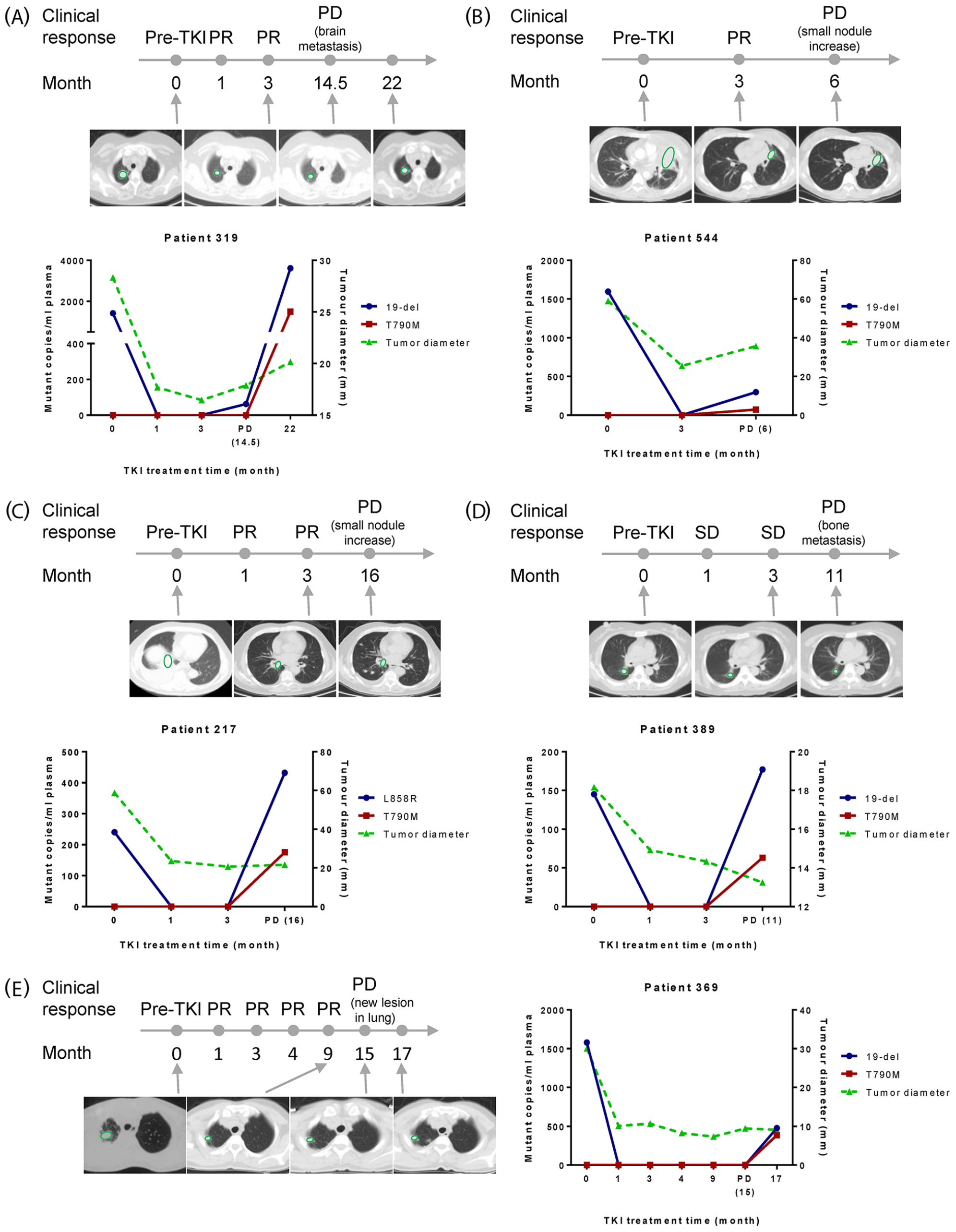

Supplementary Figure 3: Correlation of EGFR mutation abundance in plasma with development of new lesions. X-axis is TKI treatment time and Y-axis is EGFR mutant copies per ml of plasma (left) or tumor diameter (right). **(A-E)** Results from five patients to show nice correlation between plasma EGFR mutant abundance and disease progression. For patient 319, PD was defined as development of a new brain metastasis lesion. For patient 544 and 217, PD was defined as increase of the number and size of small nodules which were unmeasurable. For patient 389, PD was defined as development of a new bone metastasis site. For patient 369, PD was defined as development of a new lesion in the lung (right lobe). CT scans of selected time points were shown and lesions were indicated by green circles.

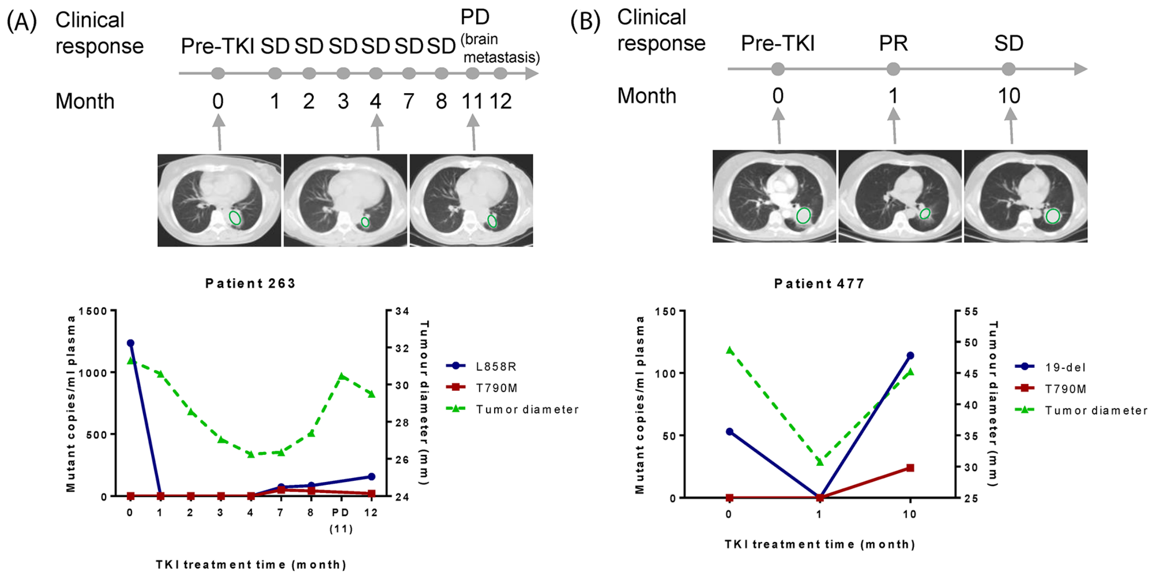

Supplementary Figure 4: Early detection of resistance by plasma EGFR mutation testing. X-axis is TKI treatment time and Y-axis is EGFR mutant copies per ml of plasma (left) or tumor diameter (right). **(A-B)** Results from two patients to show early detection of resistance before clinical PD. For patient 263, PD was defined as development of a new brain metastasis lesion. For patient 477, although clinical PD was not yet reached, a rebound in diameter of primary tumor was already observed concurrently with arise of EGFR mutations. CT scans of selected time points were shown and lesions were indicated by green circles.

Supplementary Table 1: EGFR mutation type(s) in pre-TKI plasma and tumor specimens of 45 NSCLC patients

See Supplementary Table 1

Supplementary Table 2: Dynamic detection of EGFR mutations in plasma from 45 NSCLC patients as a pool

TKI treatment time (month)	Plasma sample size	Positive sample No. (%)				
		19-del only	L858R only	L858R+19-del	L858R/19-del+T790M	T790M only
0	45	28 (62.22%)	16 (35.56%)	1 (2.22%)	0	0
1	42	6 (14.29%)	0	0	0	0
2	12	0	0	0	0	0
3	36	1 (2.78%)	0	0	1 (2.78%)	0
4, 5	18	0	0	0	0	0
6-9	23	0	1 (4.35%)	0	6 (26.09%)	0
10, 12, 17	6	0	0	0	2 (33.33%)	0
PD	20	1 (5.00%)	0	0	11 (55.00%)	0
Post-PD	8	1 (12.50%)	0	0	5 (62.50%)	1 (12.50%)

Supplementary Table 3: Dynamic monitoring of EGFR mutations in plasma from 27 NSCLC patients included in the analysis of resistance profiles or correlation with clinical response

See Supplementary Table 3

Supplementary Table 4: EGFR mutation status in plasma at 1-month post-TKI time point from 34 NSCLC patients and their PFS information

Patient ID	L858R/19-del/T790M	PFS (months)	Median PFS (months)
211	**Y (19-del)**	12.0	
220	**Y (19-del)**	7.5	
232	**Y (19-del)**	6.0	6.0
281	**Y (19-del)**	4.0	
YL10862396	**Y (19-del)**	3.0	
183	N	8.0	
185	N	15.0	
186-2	N	11.0	
187	N	10.0	
190	N	11.0	
197	N	12.0	
198	N	13.0	
201	N	19.0	
206	N	8.0	
208	N	5.0	
215	N	25.0	
217	N	16.0	
250	N	7.0	
258	N	10.0	
259	N	12.5	11.0
262	N	15.0	
263	N	11.0	
279	N	5.0	
292	N	4.5	
296	N	11.0	
303	N	16.5	
319	N	14.5	
357	N	13.0	
369	N	15.0	
389	N	11.0	
394	N	10.0	
520	N	7.0	
852957	N	12.0	
b02423525	N	7.0	
